# A Novel IEEE 802.15.4e DSME MAC for Wireless Sensor Networks

**DOI:** 10.3390/s17010168

**Published:** 2017-01-16

**Authors:** Prasan Kumar Sahoo, Sudhir Ranjan Pattanaik, Shih-Lin Wu

**Affiliations:** 1 Department of Computer Science and Information Engineering, Chang Gung University, Taoyuan 33302, Taiwan; pksahoo@mail.cgu.edu.tw; 2 Department of Electrical Engineering, Chang Gung University, Taoyuan 33302, Taiwan; d0121009@stmail.cgu.edu.tw; 3 Department of Cardiology, Chang Gung Memorial Hospital, Taoyuan 33305, Taiwan; 4 Department of Electrical Engineering, Ming Chi University of Technology, New Taipei City 24301, Taiwan

**Keywords:** wireless sensor networks, IEEE 802.15.4e, performance analysis, DSME, star topology

## Abstract

IEEE 802.15.4e standard proposes Deterministic and Synchronous Multichannel Extension (DSME) mode for wireless sensor networks (WSNs) to support industrial, commercial and health care applications. In this paper, a new channel access scheme and beacon scheduling schemes are designed for the IEEE 802.15.4e enabled WSNs in star topology to reduce the network discovery time and energy consumption. In addition, a new dynamic guaranteed retransmission slot allocation scheme is designed for devices with the failure Guaranteed Time Slot (GTS) transmission to reduce the retransmission delay. To evaluate our schemes, analytical models are designed to analyze the performance of WSNs in terms of reliability, delay, throughput and energy consumption. Our schemes are validated with simulation and analytical results and are observed that simulation results well match with the analytical one. The evaluated results of our designed schemes can improve the reliability, throughput, delay, and energy consumptions significantly.

## 1. Introduction

In Wireless Sensor Network (WSNs), devices are spatially distributed to monitor the physical or environmental conditions such as temperature, sound, pressure etc. Nowadays, WSNs are used in many industrial and consumer applications to monitor and control the industrial process, machine health, air pollution and many others. ZigBee devices are suitable to play major roles in different applications of Internet of Things (IoT). Large numbers of devices are deployed for different applications in IoT. Hence, many devices compete with each other to acquire the channel for data communications. IEEE 802.15.4 [1] is a popular standard for the medium access control (MAC) scheme of low-powered, low data rate wireless sensors. However, in order to meet the critical requirements such as low latency in industrial and commercial applications, IEEE 802.15.4e Working Group has redesigned the existing 802.15.4 MAC scheme.

IEEE 802.15.4e [2] standard defines the slotted medium access control scheme for the short-range communication devices with low data rates. There are many MAC schemes for different applications defined by IEEE 802.15.4e such as Time Slotted Channel Hopping (TSCH), Low Latency Deterministic Networks (LLDN), Deterministic and Synchronous Multi channel Extension (DSME), Radio Frequency Identification blink (RFID), and Asynchronous Multi-Channel Adaptation (AMCA). Out of these, DSME mode proposed in IEEE 802.15.4e standard [2] is designed for the habitat monitoring, process automation, factory automation, smart metering, home automation, smart building, entertainment, patient monitoring and telemedicine applications. The MAC scheme of DSME superframe structure extends the number of GTS slots and the single channel operation of IEEE 802.15.4 standard to multichannel operation for data transmission. DSME superframe structure supports the star topology based WSNs, where collisions occur either due to hidden terminal problem or simultaneous channel assessment for data transmission.

Hidden terminal problem can be avoided by allocating a common Contention Access Period (CAP) in the superframe to the devices present within the same communication range. CSMA/CA scheme avoids the collision during the transmission of other devices, which are not hidden. In WSN, large numbers of devices are deployed for different applications in which many devices attempt to send frames simultaneously resulting significant collisions in the system. The probability of collisions is related directly to the number of devices in the network and this issue is significant, when large number of devices are associated with one coordinator. To fulfill the present need of different applications, the coordinator needs to support more devices, which may result higher rate of collisions. The performance of CAP is deteriorated when large numbers of devices contend with each other.

In IEEE 802.15.4e MAC, nodes perform slotted carrier sense multiple access with collision avoidance (CSMA/CA) mechanism to access the channel and uses a random backoff algorithm to reduce the collision probability. To save power consumption, the devices use power saving mode during the backoff duration and perform carrier sensing after the backoff duration is over. The channel access scheme suggested by IEEE 802.15.4e standard simply adopts a random backoff with clear channel assessment (CCA). The channel busy occurs either due to data or acknowledgement transmission. However, the current scheme proposed in IEEE 802.15.4e increases delay as well as energy consumption and reduces the throughput of the network due to inefficient backoff and carrier sensing management. Hence, this problem needs to be addressed to reduce unnecessary backoff and carrier sensing.

The DSME MAC supports a group acknowledgement option to provide a retransmission opportunity within the same superframe for any data frame, which is failed in its DSME-GTS transmission. This group acknowledgement option improves the network efficiency as acknowledgement (ACK) for multiple data frames are aggregated to a single ACK frame. However, the devices allocate an additional DSME GTS for Retransmission (GTSR) per each allocated DSME GTS to transmit data to the coordinator. These slots are used for retransmissions of their failed GTS transmissions or for transmitting additional data frames. If a device is succeeded in the first attempt of the GTS and has no new data to transmit in the allocated DSME-GTSR, the allocated slot becomes useless. This problem needs to be addressed so that those DSME-GTSR slots can be reused for other devices.

Mobility of devices are imminent in many IoT applications such as habitat monitoring, home automation, smart building, entertainment and patient monitoring etc. Due to mobility, devices frequently leave or enter into the network, by which their synchronization is lost. In order to join a beacon enabled network, devices first discover the coordinator by scanning channels and then join the network by using association messages. The channel scanning durations are defined based on the Beacon Order (BO) of the coordinator. The longer is the BO, the higher is the latency of the coordinator discovery. Again, neither the channel on which the coordinator operates nor its frequency of beaconing is known to a new device that enters into the network. Therefore, minimization of association time for a device with the coordinator is a challenging task, which needs to be addressed.

### Motivation

In a WSN, normally nodes are deployed densely around the coordinator and several nodes may compete with each other to transmit data simultaneously. Though, IEEE 802.15.4e has its own collision avoidance mechanism, there is no provision to reduce the probability of collisions by restricting the number of devices to be associated with the coordinator at the same time. Hence, it is essential to restrict large numbers of devices to be associated with the coordinator or none of devices can get benefits to transmit data. In IEEE 802.15.4e medium access mechanism, a node has to go for clear channel assessment (CCA) after its random backoff period is over. It senses the channel busy if any other node within its transmission range is transmitting either data or ACK and therefore it increases the delay and consume more power due to another longer backoff. It is necessary to devise some mechanism by which unnecessary backoffs can be avoided. Hence, we design a new carrier sensing mechanism by which a node can avoid sensing the channel busy situation when the exchange of ACK is going on by other node.

The group ACK option given in DSME MAC of IEEE 802.15.4e provides a retransmission opportunity to the nodes within the same superframe. Accordingly, an additional DSME GTS is allocated for Retransmission (GTSR) to the coordinator. However, these allocated slots become useless, if a device is succeeded to transmit data in its first attempt and has no new data to transmit. Therefore, we design here one dynamic GTS allocation scheme by which the retransmission delay can be reduced and the network throughput can be improved. In the beacon enabled IEEE 802.15.4e network, devices first discover the coordinator by scanning the channels. The easiest way for coordinator discovery is to scan all channels sequentially with maximum beacon interval, which is approximately 250 s. Hence, discovery of a beacon by scanning 16 channels would take an average of 33 min (250 s × 16/2). In order to mitigate this delay, we design a novel MAC mechanism for the IEEE 802.15.4e enabled WSNs and analyze its performance. The main contributions of our work can be summarized as follows.

- A new contention channel access scheme is designed for wireless sensors to enhance the throughput, reliability and to reduce the power consumption.
- A new dynamic GTS allocation scheme is designed to reduce the delay for the devices who have failed to transmit data.
- A new beaconing scheme is designed to reduce the association delay for orphan mobile devices.
- A Markov chain model is designed for the performance analysis of the system in terms of delay, throughput, reliability and energy consumption due to data transmission.

Rest of the paper is organized as follows. Related works of the existing IEEE 802.15.4e standard are given in Section 2. Network model and proposed methods are presented in Section 3. Analytical models are given in Section 4. Performance analysis of different network parameters based on our models are given in Section 5. Simulation results are presented in Section 6 and concluding remarks are made in Section 7.

## 2. Related Works

Many channel access schemes have been proposed to enhance IEEE 802.15.4 performance for different performance parameters like throughput, delay, reliability, and energy consumption. A generalized approach to analyze the performance of the slotted CSMA/CA scheme in the IEEE 802.15.4 standard is designed in [3]. Authors in [4] present an analytical model for the MAC scheme of IEEE 802.15.4 to predict the energy consumption as well as the throughput of the saturated and unsaturated traffic rates. A Markov chain based analytical model is introduced in [5] to evaluate the impact of throughput and energy consumption on the probability of delivering a packet. An integrated cross-layer model of the MAC and physical layers for unslotted IEEE 802.15.4 networks is presented in [6] by considering explicit effects of multi-path shadow fading channels in the presence of interferers. In an event driven application, all the associated devices start transmitting data simultaneously in non beacon enabled wireless sensor network. Reference [7] provides an accurate performance analysis for event-driven CSMA/CA WSNs. Authors in [8,9] have designed the time critical services and priority based adaptive MAC schemes for the WSNs. However, these schemes do not talk how to increase reliability of the network.

An analytical model based on a Markov chain for the GTS allocation scheme in the contention-free period (CFP) is presented in [10]. Authors in [11,12] have designed the GTS allocation scheme based on the data size and mobility of the devices for an optimal utilization of the channel bandwidth. In a wireless network due to some hardware failure, devices associated with particular application start sending faulty data. Again limited link capacity forces the devices to send the quantized data. Under such scenario to enhance the reliability authors in [13] propose a multiple superframe structure and a non-preemptive GTS scheduling algorithm. In a wireless network, devices synchronously alternate active and sleep modes and completely turnoff the radio during sleep mode. Authors in [14] provide comprehensive review synchronous MAC schemes with respect to latency. However, authors in these papers have not considered the retransmission opportunity for the transmission failure during GTS.

A significant amount of packet loss occurs due to interference, which degrades the expected performance in wireless network. To avoid Wi-Fi interference among the devices in a network, devices should use orthogonal channels. However, limited number of orthogonal channels are available. Hence, authors in [15] propose one frequency agility based interference avoidance algorithm that detects the interference and devices switch to the safe channel adaptively. Authors in [16] perform theoretical analysis of throughput for WSNs under external interference with deployment of forward error correction. Also, authors in [17] have studied different types of interferences, direction of signal arrival and attenuation losses associated with smart building environment. However, none of the works has considered the reliability of the data communications.

An analytic model is designed in [18] to study the MAC performance and reliability. Energy is a major constraint for device and the major energy consumption of device is due to transmission. As channels are considered with temporal and fixed path loss values in body area networks, authors in [19] have suggested through experimental results that transmitting in −12 dBm to −15 dBm is suitable for energy efficient body area networks. A comprehensive analysis of energy consumption of body area networks including the effect of packet inter-arrival time is given in [20]. Using the neighbors information regarding packet size, data interval, energy and consumed bandwidth, a new MAC scheme is proposed in [21] to minimize the energy consumption in wireless sensor network. Authors in [22] propose a priority-based adaptive time slot allocation scheme for the IEEE 802.15.4 MAC scheme. On the other hand, these schemes are based on ideal channel condition only and are not realistic.

An analytical model is designed in [23] to predict the throughput and energy consumption of the IEEE 802.15.4 networks under non-saturated wireless environment. Authors in [24] have designed one additional carrier sensing (ACS) algorithm to get the information of busy channel due to data or an acknowledge packet transmission during the second CCA. However, it is wastage of energy if the second CCA is found busy due to data. In order to increase the chance of transmission by ignoring the channel busy condition due to end of ACK packet transmission during first CCA, authors in [25] propose one segmentized CCA. However, it has no improvements for the data transmission, if it is a data packet instead of ACK one. In order to avoid collision in a dense network, a new variable Clear Channel Assessment MAC scheme for WSNs along with performance analysis is designed in [26]. However, these schemes consumes significant amount of energy for CCAs. A mathematical model for deriving the energy consumptions in clustered IEEE 802.15.4 WSNs is designed in [27], which is suitable only for non beacon-enabled WSNs.

Performance study of sending and receiving capabilities of commonly used devices in wireless network are made in [28]. Though, they investigate the communication performances of various devices, data reliability, energy consumption and other related performance metrics have not been studied in their work. Authors in [29] have experimentally analyzed the aggregate throughput with different traffic loads and under interference of IEEE 802.11b. Performance of IEEE 802.15.4e Time Slotted Channel Hopping (TSCH) and DSME have been studied in [30] to analyze the energy consumption of the system. However, none of these work proposes new MAC mechanism for the IEEE 802.15.4e DSME. Besides, none of the work also analyze the performance in terms of reliability and latency. A discrete-time Markov chain model is used in [31] to analyze the throughput of IEEE 802.15.4 MAC scheme in presence of the hidden devices. Most of the existing analytical models of IEEE 802.15.4 MAC scheme assume ideal channel conditions. However, wireless channels exhibit burst errors in real world scenarios. A three-dimensional discrete-time Markov Chain model is proposed in [32], which is applied to analyze the performance of IEEE 802.15.4 MAC scheme under burst channel errors. In [33], authors evaluate the impact of losing synchronization in beacon-enabled IEEE 802.15.4 network with star topology. However, they did not considered how to reduce synchronization time for orphan or mobile devices. To study the aggregate network throughput and packet delivery delay, authors in [34] propose an analytical framework. However, they do not consider the transmission reliability. From the survey of the current literature, it is observed that most of the performance analysis are based on the IEEE 802.15.4 standard.

In typical WSN applications such as smart home, smart industrial automation and smart health care, all devices are connected to a coordinator, which is a single hop scenario. It can be extended to multihop networks by considering the communications among coordinators. In this paper, we focus on how to optimize the performance of the single hop network. This paper not only proposes the DSME MAC schemes for single hop WSN, but also designs the analytical models to evaluate the performance in terms of maximum throughput, delay, energy consumption and reliability based on the IEEE 802.15.4e instead of IEEE 802.15.4 standard.

## 3. The Proposed DSME MAC

In order to avoid collision due to simultaneous transmissions, a new channel access scheme is designed for the IEEE 802.15.4e star topology WSNs. The network model and the proposed schemes are illustrated as follows.

### 3.1. Network Model

**Figure 1:**
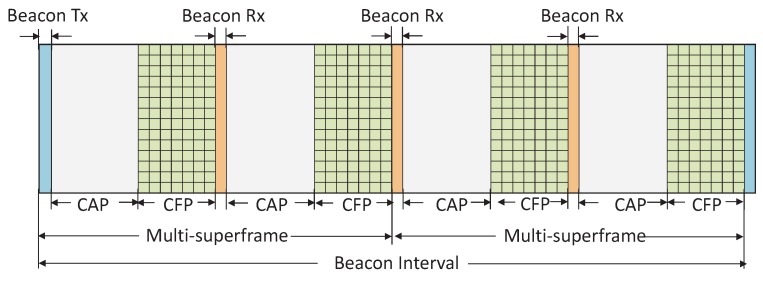
DSME superframe structure with BO = 6, MO = 5, SO = 4.

Let us consider a beacon enabled star topology of IEEE 802.15.4e based WSNs with *N* numbers of devices as $S_{1},S_{2},\ldots .,S_{N}$, which are connected to a PAN coordinator. It is assumed that each device follows the DSME superframe structure of IEEE 802.15.4e as shown in Figure 1.

The CSMA/CA scheme is the key component in IEEE 802.15.4e MAC. It is adopted to arrange the devices in the network with an appropriate order when they access the channel. All devices use CSMA/CA channel access scheme in the CAP period to transmit data frames to the coordinator. Every device will go for the power saving mode after packet transmission. When a device access the channel in the CAP period, it may defer transmission, if the remaining slots are not enough to transmit the data.

### 3.2. A New Association Scheme

In this section, a new scheme of beaconing by the PAN coordinator is designed for the fast association of the devices with it. As per the IEEE 802.15.4e standard, the numbers of superframes, multisuperframes present in a DSME beacon interval are $2^{BO-SO}$ and $2^{BO-MO}$, where BO, SO, MO are macBeaconOrder, macSuperframeOrder and macMultisuperframeOrder respectively. As shown in Figure 1, if the coordinator chooses BO=6, MO=5 and SO=4, the number of superframes $2^{BO-SO}$ is 4. Each superframe has one exclusive beacon slot and therefore the total numbers of available beacon slots in the DSME superframe are $2^{BO-SO}$. According to the current standard, coordinator transmits a beacon only at the start of DSME superframe in one selected channel. In order to reduce the discovery/association delay, we propose that the coordinator should broadcast the beacon in different channels during beacon slots. Let $CH=\{CH_{1},CH_{2},\ldots .CH_{k}\}$ be the set of channels and $SUP=\{SF_{1},SF_{2},\ldots .SF_{2^{BO-SO}}\}$ be the set of superframes present in the DSME beacon interval. According to our proposed scheme, the coordinator broadcasts the beacon in the *i*th channel, i.e., in $CH_{i}$ and *j*th superframe, i.e., in $SF_{j}$.

(1) $$\begin{array}{cc} (j+BN*2^{BO-SO})\equiv i\;MOD\;k & \;for\;1\leq i\leq k\;and\;1\leq j\leq 2^{BO-SO} \end{array}$$

where the variable BN represents the beacon sequence number.

When a new device enters into the network and wants to communicate, it should be associated with the PAN coordinator. To perform association, the device must perform the channel scanning to receive a beacon broadcast by the coordinator. The device keeps its radio ON during the scanning until it receives a beacon. There are two types of scanning procedures (active and passive) suggested in the IEEE 802.15.4e standard. In active scanning, devices have to send beacon request command in the chosen channel, which is an energy consuming process. In passive scanning, devices have to listen to the chosen channel until they locate the coordinator. However, passive scanning to discovery the coordinator increases the delay. Since, power consumption is a major constraint in WSN, we focus on passive scanning scheme to save the power. In our proposed method, the new device that wants to join the network should choose a random channel and perform the passive scan, i.e., it waits for the beacon in that channel. The device definitely gets the beacon as the coordinator broadcasts the beacon in every channel. Upon receiving the beacon frame, the devices send the association request frame using CSMA/CA and the coordinator sends back the association response frame, if it has received the association request successfully. Otherwise, the devices repeat the association procedure as suggested in the IEEE 802.15.4e standard.

In our proposed scheme, un-associated devices can chose one random channel and perform the passive scan. Hence, our scheme can reduce the busy channel conditions and collisions when un-associated devices are increase. However, according to the IEEE 802.15.4e, all un-associated devices can locate the coordinator simultaneously through the passive scan and all of them can have access to the channel simultaneously. This procedure can increase the delay for the discovery of the coordinator and association with it. On the other hand, our procedure can significantly reduce the delay for discovering the coordinator and association with it. The detailed procedure of our proposed schemes are given in Algorithm 1 and Algorithm 2 for the coordinator and devices, respectively.
**Algorithm 1** A new beaconing scheme for coordinator.**Require:** Set of channels $CH=\{CH_{i}|1\leq i\leq k\}$, beacon order (BO), superframe order (SO) and beacon sequence number (BN).**Ensure:** Beacon broadcast in every channel.
1:$l=2^{BO-SO}$;2:Set of superframes $SUP=\{SF_{j}|1\leq j\leq l\}$;3:**for** (1≤i≤k) **do**4:  **for** (1≤j≤l) **do**5:    **if** ((j+BN×l)MODk=i) **then**6:      Broadcast beacon for superframe $SF_{j}$ in channel $CH_{i}$;7:    **end if**8:  **end for**9:**end for**
**Algorithm 2** A new association scheme for device.**Require:** Set of channels $CH=\{CH_{i}|1\leq i\leq k\}$.**Ensure:** Successful association with a suitable coordinator.
1:Generate a random number *r* = random (1, k);2:Passive scan in channel $CH_{r}$.3:**if** Beacon received **then**4: **repeat**5:   Compete with other devices to associate with the coordinator;6: **until** Successful association.7:**else**8: Wait for beacon;9:**end if**


### 3.3. A New Channel Access Scheme

CSMA/CA scheme is the key component in IEEE 802.15.4e MAC. It is adopted to arrange the devices in the network with an appropriate order when they access the channel. When too many devices have data to transmit to the coordinator at the same time, they try to access the channel simultaneously, which increases the collision and makes the channel busy. As per IEEE 802.15.4e standard, the numbers of superframes present in the DSME superframe are $2^{BO-SO}$. Hence, the number CAP period presented in the DSME superframe will be $2^{BO-SO}$. To restrict the number of users to access the channel simultaneously, we propose here a new channel access scheme in which each device is allowed to contend the channel only in one CAP period.

Upon receiving the beacon successfully, each device knows the value of BO and SO from the beacon and gets the numbers of CAP period presented in the DSME superframe as $2^{BO-SO}$. Based on the following equation, each device calculates the index of its assigned CAP period.

(2) $$\begin{array}{c} CAP_{index}=\left(S_{ID}+N_{offset}\right)MOD\left(2^{BO-SO}\right) \end{array}$$

where $S_{ID}$ is ID of each device and $N_{offset}$ represents the offset value in the mapping function. $N_{offset}$ is determined based on a time stamp divided by a beacon interval field in a beacon frame from the coordinator. It is to be noted that $N_{offset}$ improves the fairness among the devices as it is changed with respect to the beacon interval. Hence, the mappings of devices to the CAPs are different among beacon intervals. All devices upon receiving the beacon are synchronized and go for the power saving mode. The devices wake-up at the start of the assigned CAP period to access the channel. The devices those are assigned to a CAP period contend with each other in that CAP period only. Our proposed mapping function can allocate the devices in to different CAPs so that the collision rate can be reduced significantly.

**Figure 2:**
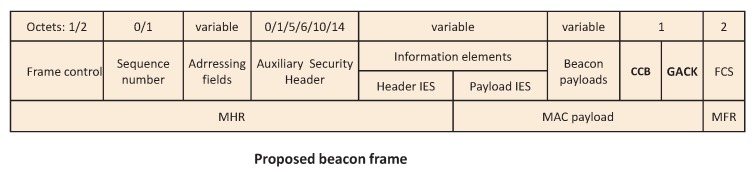
A new beacon frame for IEEE802.15.4e.

After the allocation, the variables NB, CW, BE and RT are initialized for each allocated device. NB is the number of times the CSMA/CA scheme required to delay while attempting the current transmission, which is initialized to 0 before every transmission. CW is the contention window length and BE is the backoff exponent. RT is the maximum number of retransmission attempts. Each device generates a random backoff counter $\left(R_{b}\right)$ in the range of $\left[0;2^{BE}-1\right]$ units. When the backoff period is count down to zero, the tagged device performs the CCA if the remaining time $R_{CAP}$ in the CAP period is enough to perform the CCA and to transmit the required data. Let the duration of one backoff slot, time duration to perform one CCA, time for data transmission be *σ*, $T_{CCA}$ and $T_{D}$, respectively. It is to be noted that the CAP and CFP period in the superframe is fixed. According to the standard, GTS slots are allotted to different devices based on their requests received by the coordinator during CAP. However GTS slots may be under utilized, if coordinator receives less number of requests as compared to the available slots in the CFP, which will affect the network throughput. Hence, we modify the beacon format by adding one bit field called Cross CAP Boundary (CCB) as shown in Figure 2 to utilize the unused slots, which is allowed by the standard.

**Figure 3:**
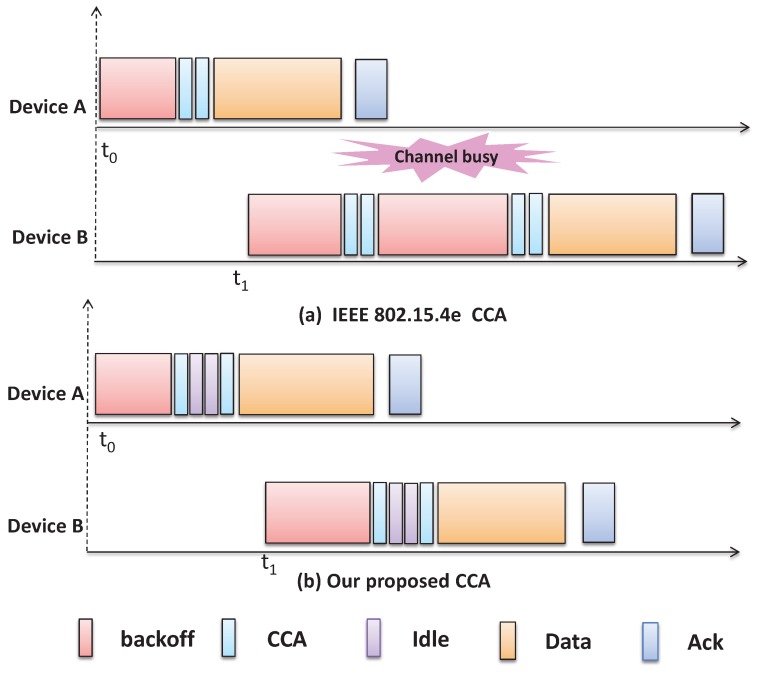
A new CCA scheme for IEEE802.15.4e to avoid channel busy due to acklodgement.

As per the IEEE 802.15.4e standard, it is to be noted that a device doubles the contention window size up to $MAX_{NB}$ and performs the CCA again by choosing a random delay within the contention window and discards the packet after maximum number of retransmission $MAX_{RT}$ attempts if the device finds the channel busy during any of the CCA. After finding the channel idle in the first CCA, the second CCA is performed and transmission is started if the channel is found idle. This procedure is repeated if the channel is found busy during the second CCA, which may be incurred by two reasons. When a tagged device performs the first CCA if another device performs its second CCA, the channel remains busy. Also the channel remains busy, if the tagged device performs the first CCA during the acknowledgment waiting time $T_{ACK}$ of another device. One solution to reduce the channel busy is to increase the duration of the backoff period of a sensor. However, by doing so, the network performance like transmission delay will be increased and fairness may not be maintained. Besides, it could be possible that a node may still assess the channel busy during the transmission of ACK by other nodes. For example, as shown in Figure 3, node *B* assesses busy channel, when node *A* transmits the ACK packet to the coordinator. If the backoff period is increased, it will be applicable for all devices and accordingly, node *B* can have same problem, when the ACK transmission is going on in the medium. However, our proposed mechanism can avoid this problem as a node has to adopt two idle slots after its first CCA and by that time ACK transmission if any can be completed.

The tagged device can transmit the data packet and waits for the acknowledgment, if the channel is found idle during the second CCA. After the $T_{ACK}$ time is out, if the device does not receive the acknowledgment, it assumes that collision might have occurred in the channel and therefore repeats the procedure. The detailed procedure of our proposed scheme is given in Algorithm 3.
**Algorithm 3** New channel access scheme.**Require:**
$NB=0,BE=macMinBE,RT=0,\sigma =20\;symbols,T_{CCA}=8\;symbols$ , CCB=0or1 and packet duration time $T_{D}$.**Ensure:** Transmission success/failure.
1:$\Delta =2\times T_{CCA}\;+\;2\times \sigma \;+\;T_{D}$2:**if** Beacon received **then**3: Calculate the $CAP_{index}$ and wakeup at start of the $CAP_{index}$;4:**else**5: Wait for beacon;6:**end if**7:Generate a random backoff time $R_{b}$ = $random(0,\;2^{BE-1})\times \;\sigma$;8:**if** $(\left(R_{CAP}\leq \Delta \right)$ or CCB=0) **then**9: Wait for next superframe and go to step 2;10:**else**11: Perform CCA1 after $R_{b}$ duration;12: **if** Channel found busy **then**13:  Switch to sleep state for the $random(0,T_{D})$ and go to step 30;14: **else**15:  Wait for 2σ and perform CCA2;16:  **if** Channel found busy **then**17:   Switch to sleep state for $T_{D}$ and go to step 30;18:  **else**19:   Start transmission;20:   **if** Transmission failure **then**21:    RT=RT+1;22:    **if** RT ≤ $MAX_{RT}$ **then**23:     Go to step 7;24:    **else**25:     Transmission failure;26:    **end if**27:   **end if**28:  **end if**29: **end if**30: NB=NB+1, BE=min(BE+1,macMaxBE);31: **if** $\left(NB>MAX_{NB}\right)$ **then**32:  Transmission failure;33: **else**34:  Go to step 7;35: **end if**36:**end if**


According to IEEE 802.15.4e enabled star topology, all devices need to be associated with a single coordinator. Accordingly, all devices can have right to access the channel simultaneously and, as a result, collisions may occur. However, in our proposed scheme, devices are distributed to different superframes during their channel access mechanism so that less number of devices can access to the channel as compared to the IEEE 802.15.4e standard and therefore can minimize the collisions. Thus, our proposed mechanism can be suitable even in the large scale network.

### 3.4. Proposed Dynamic GTS Allocation Scheme

Group acknowledgement (GACK) plays an important role to provide the transmission opportunity within the same DSME superframe. The failed devices who get the information in GACK can have a chance to transmit data again in the allotted slots within the DSME superframe. In the current IEEE 802.15.4e standard, the coordinator allocates two separate slots within the CFP for this propose. Again the device has to reserve two slots, i.e., GTS and GTSR for each transmission slot in order to avail the retransmission opportunity within the DSME superframe. The next GTSR allotted slot will be used for new data packet of that device, if the transmission is successful in the first GTS allotted slot. Most of the time the devices may not have new data to send in the GTSR allotted slots. Hence, the overall throughput may be reduced significantly under this scenario.

To overcome the drawbacks of the GTS scheme of IEEE 802.15.4e standard, we design here a new low complexity and reliable GTS allocation scheme. In order to increase the network throughput and to reduce the delay for retransmission, we modify the beacon format by adding a new seven bit field to the beacon frame called GACK as shown in Figure 2. The CFP of one superframe contains seven GTS slots and these seven bits will be one or zero based on the corresponding GTS transmission is success or failed, respectively. Upon receiving the beacon, each device counts the number of bits having value zero among these seven bit field and starts the channel access during CAP after those number of slots. The failed devices find the suitable slot during the CAP based on the received seven bit field and transmit again without following the CSMA/CA procedure. Hence, the delay can be minimized and unnecessary energy consumption due to contention in the CAP can also be reduced.

**Figure 4:**
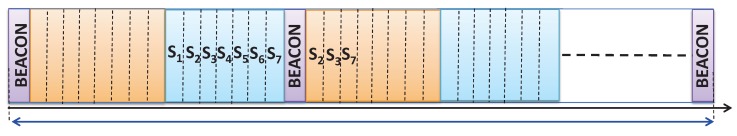
Dynamic GTSR allocation for the failed GTS transmission.

As shown in Figure 4, let devices $S_{1},S_{2}\ldots ,S_{7}$ be allotted to GTS slots 1, 2, ..., and 7, respectively. It is assumed that devices $S_{2},S_{3},S_{7}$ fail to transmit the data during their allotted 2nd, 3rd and 7th GTS slots, respectively due to interference or fading or channel error. The coordinator will inform the transmission failure through the GACK field present in the beacon frame as shown in Figure 2. The value in the 2nd, 3rd and 7th bit of the GACK field is 0. The superframe presents in a DSME superframe contains 16 slots out of which one slot is a beacon slot, next eight (1–8) slots are CAP and the rest seven (9–15) slots are CFP. devices those who receive the beacon get the information about these failed transmission by counting the number of zero bits in the GACK field and start the channel access from the 4th slot of the current CAP. The failed devices $S_{2},S_{3},S_{7}$ will start retransmission at slot number 1st, 2nd and 3rd, respectively in CAP without contention.

According to the existing mechanism of IEEE 802.15.4e, failed devices has to wait for the next retransmission slots to be allocated in the GTS. However, as shown in Figure 2, we add a GACK slot in our proposed MAC so that the failed devices neither have to contend for the channel nor have to wait for the next allotted retransmission slots. Besides, the proposed scheme can have the same superframe structure defined by the IEEE 802.15.4e standard.

## 4. Analytical Models for DSME MAC

In this section, we propose the Markov model to analyze the channel access scheme of the devices in the shared slots. It is assumed that the traffic condition is unsaturated. Based on such unsaturated traffic condition and the notation given in Table 1, the analytical model can be designed as follows.

**Figure 5:**
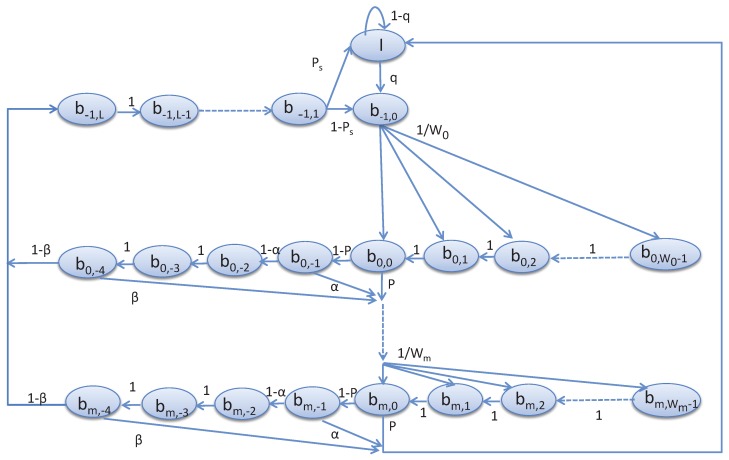
Markov model for the proposed CSMA-CA scheme.

We assume that *M* devices are allotted to one superframe for data communication with the coordinator. For a given device, the stochastic processes s(t) and c(t) represent the backoff stage for *NB* and backoff counter for *CW*, respectively as shown in Figure 5. Let

$$b_{i,x}=\lim_{t\to \infty }P\left\{s\left(t\right)=i,c\left(t\right)=x\right\},$$

where $x\in \{-1,\ldots ,W_{i}-1\}$ $W_{i}=2^{min(i+macMINBE,macMAXBE)}$i∈{0,…,m}. The time *t* corresponds to the beginning of the slot time and is directly related to the system time. $b_{i,-1}$ represents the states corresponding to the start of the channel access, where i∈{0,…,m}. In our our proposed model, since a device has to wait for two backoff slots before performing the second CCA, we represent the states $b_{i,-2}$ and $b_{i,-3}$ corresponding to the waiting states prior to the second CCA, where i∈{0,…,m}. $b_{i,-4}$ represents the states corresponding to the second CCA before start for transmission, where i∈{0,…,m}. $b_{-1,l}$, represents the transmission state, where l∈{1,2,…,L}. *L* is the number of transmission slots. The state *I* stands for the idle state when a device has no request packet to transmit. *q* is defined as the probability that the device has a packet to transmit. After generating the request packet, a device goes to the state $b_{-1,0}$. Upon reaching at the state $b_{-1,0}$, the device chooses a random backoff and goes to the state $b_{i,0}$, where $i\in \{0,1,2\ldots ,W_{0}-1\}$. The state $b_{i,j}$ represents the backoff states, where $i\in \left\{0,\ldots ,m\right\},\;j\in \left\{-1,\ldots ,W_{i}-1\right\}$.

Let, *α* and *β* be the probability of accessing channel busy during the first and second *C*CA, respectively. Let *p* be the probability that the remaining time in the CAP period is sufficient to complete the data transmission. A device goes to the transmission state, if the channel is idle and attempts to transmit requests. Let $P_{S}$ be the probability that the transmission is successful.

Based on the proposed Markov chain model given in Figure 5, the steady state probabilities can be derived as follows.

(3) $$\begin{array}{c} P\left\{b_{i,k}|b_{i,k+1}\right\}=1\;\;for\;0\leq i\leq m\;and\;0\leq k\leq W_{i}-1. \end{array}$$

(4) $$\begin{array}{c} P\left\{b_{i,k}|b_{i-1,0}\right\}=\frac{p+(1-p)\alpha +(1-p)(1-\alpha )\beta }{W_{i}}\\ \;for\;0\leq i\leq m\;and\;0\leq k\leq W_{i}-1. \end{array}$$

(5) $$\begin{array}{c} P\left\{I|b_{m,0}\right\}=\left(p+\left(1-p\right)\alpha +\left(1-p\right)\left(1-\alpha \right)\beta \right)+\left(1-p\right)\left(1-\alpha \right)\left(1-\beta \right)P_{S}. \end{array}$$

Equation (3) represents the decrement of backoff counter, which occurs with probability 1. Equation (4) represents the probability of finding channel busy during CCA and thereafter a device selects uniformly a state in the next backoff state. Equation (5) gives the probability of going back to the idle state from the last backoff state after finding channel idle or busy during the CCA. Based on these transition probabilities, we can derive the steady state probabilities as follows. The steady state probability that a device enters into the next backoff state after the channel access failure in the current backoff state is

(6) $$\begin{array}{c} b_{i,k}=\frac{W_{i}-k}{W_{i}}\left[b_{i-1,0}\left\{p+\left(1-p\right)\alpha +\left(1-p\right)\left(1-\alpha \right)\beta \right\}\right];\\ \;\;\;for\;1\leq i\leq m\;and\;0\leq k\leq W_{i}-1. \end{array}$$

It is to be noted that a tagged device starts transmission upon accessing to a channel successfully. However, if the transmission is successful or failed without exceeding the retry limits, it will again enter into the initial backoff state. Hence, the corresponding steady state probability is

(7) $$\begin{array}{c} b_{0,k}=\frac{W_{0}-k}{W_{0}}[\left\{\sum_{i=0}^{m}b_{i,0}\right\}\left(1-p\right)\left(1-\alpha \right)\left(1-\beta \right)\left\{\left(1-P_{S}\right)+P_{S}q\right\}\\ +b_{m,0}\left(p+\left(1-p\right)\alpha +\left(1-p\right)\left(1-\alpha \right)\beta \right)q];\;\;\;0\leq k\leq W_{i}-1. \end{array}$$

Normally, a device goes for CCA after the random backoff period and the remaining time in the CAP period is sufficient for the data transmission. Therefore, the corresponding steady state probability can be deduced as follows.

(8) $$\begin{array}{cc} b_{i,-1} & =\left(1-p\right)b_{i,0};\;\;\;\mathrm{for}\;0\leq i\leq m. \end{array}$$

Normally, a device goes for the second CCA, if the channel is found idle during the first CCA. Hence, the corresponding steady state probability can be derived as follows.

(9) $$\begin{array}{cc} b_{i,-y} & =\left(1-\alpha \right)b_{i,-1};\;\;\;\mathrm{for}\;0\leq i\leq m\;\mathrm{and}\;-4\leq y\leq -2. \end{array}$$

After accessing the channel successfully in the $i^{th}$ backoff state, the device starts transmitting the data and the corresponding steady state probability is given as follows.

(10) $$\begin{array}{cc} b_{-1,l} & =\left(1-\beta \right)b_{i,-4};\;\;\;\mathrm{for}\;0\leq i\leq m\;\mathrm{and}\;\;\;\mathrm{for}\;1\leq l\leq L. \end{array}$$

A device that enters to the idle state either completes the data transmission or has no packet to transmit. Hence, the corresponding steady state probability is

(11) $$\begin{array}{cc} I & =\frac{1}{q}\left(P_{S}b_{-1,1}+\left(p+\left(1-p\right)\alpha +\left(1-p\right)\left(1-\alpha \right)\beta \right)b_{m,0}\right). \end{array}$$

If a device has new packet or needs to retransmit the unsuccessful packet, it enters to the active state. The corresponding steady state probability is

(12) $$\begin{array}{cc} b_{-1,0} & =\left(1-P_{S}\right)b_{-1,1}+qI. \end{array}$$

According to the rule of the probability, i.e., sum of the probabilities of all states should be equal to 1, we can get the following equation.

(13) $$\begin{array}{cc} I+b_{-1,0}+\sum_{i=0}^{m}\sum_{j=0}^{W_{i}-1}b_{i,j}+\sum_{x=1}^{4}\sum_{i=1}^{m}b_{i,-x}+\sum_{l=1}^{L}b_{-1,l} & =1 \end{array}$$

By solving these equations, we can get the probabilities of all states. Then, the probability that a device attempts the CCA for the first time within a slot for transmitting data can be calculated as follows.

(14) $$\begin{array}{c} \phi =\sum_{i=0}^{m}b_{i,0}\left(1-p\right). \end{array}$$

## 5. Performance Analysis

The probability of the bit error rate $P_{b}\left(\zeta \right)$ for the transceiver of IEEE 802.15.4 radios is calculated [1] as given in the following equation.

(15) Pb(ζ)=815116∑k=216(−1)k16ke20ζ(1k−1)

where, *ζ* is the signal-to-interference-plus-noise ratio (SINR). Let, $L_{D}$ be the length of the data packet and $L_{Ack}$ be the length of the acknowledgement packet. Let, *q* be the probability that a device has a packet to transmit and $\alpha_{1}$ be the probability that the first CCA is busy due to data transmission by other devices. A device starts transmission, if the channel is found ideal. Hence,

(16) $$\begin{array}{c} \alpha_{1}=L_{D}\left(1-{\left(1-\phi \right)}^{M-1}\right)\left(1-\alpha \right)\left(1-\beta \right) \end{array}$$

Coordinator will send the acknowledgement packet, if it receives the packet without any collision or channel error. In other words, we can say if exactly one device transmits without any channel error, then the coordinator will send the acknowledgement packet. Let $\alpha_{2}$ be the probability that the first CCA is busy due to transmission of acknowledgement by the coordinator. Hence,

(17) $$\begin{array}{c} \alpha_{2}=M\phi {\left(1-\phi \right)}^{M-1}\left(1-\alpha \right)\left(1-\beta \right){\left(1-P_{b}\left(\zeta \right)\right)}^{L_{Data}} \end{array}$$

where, $L_{Data}$ is the length of the data packet in bits. Let, *α* be the probability that the first CCA is busy. During the first CCA, the device will find the channel busy due to the transmission of data or acknowledgement. Hence, the probability of finding the channel busy during the first CCA is given as follows.

(18) $$\begin{array}{c} \alpha =[L_{D}\left(1-{\left(1-\phi \right)}^{M-1}\right)+M\phi {\left(1-\phi \right)}^{M-1}{\left(1-P_{b}\left(\zeta \right)\right)}^{L_{Data}}]\left(1-\alpha \right)\left(1-\beta \right). \end{array}$$

Let *β* be the probability that the second CCA is busy. A device will perform the second CCA, if the channel is found ideal during the first CCA. A device transmits the data, if the channel is found idle after the second CCA. In our scheme, the channel will be busy during the second CCA due to data transmission as the acknowledgement packet is avoided by keeping two ideal slots between two CCAs. Hence, the tagged device will find the channel busy during its second CCA, if and only if any other device completes his second CCA and starts transmitting data. Hence,

(19) $$\begin{array}{c} \beta =3(1-{\left(1-\phi \right)}^{M-1})\left(1-\beta \right). \end{array}$$

The device will go for the next backoff, if the channel access fails. Hence, the probability of going to the next backoff is

(20) $$\begin{array}{c} P_{backoff}=p+\left(1-p\right)\alpha +\left(1-p\right)\left(1-\alpha \right)\beta . \end{array}$$

### 5.1. Reliability

A transmission is successful if exactly one device is transmitting without any collision and channel error. A collision occurs if at least one of the remaining devices starts sensing the channel in the same slot and transmits data upon finding the channel idle. Hence, the probability of collision is

(21) $$\begin{array}{c} P_{Collision}=1-{\left(1-\phi \right)}^{M-1} \end{array}$$

where, *M* is the number of devices. A transmission will be successful, if the device receives the acknowledgement packet corresponding to its transmission. The coordinator sends the acknowledgement, if exactly one device transmits data without any channel error. Therefore, the probability of successful transmission by a device is

(22) $$\begin{array}{cc} P_{S} & =\frac{M\phi {\left(1-\phi \right)}^{M-1}}{\left(1-{\left(1-\phi \right)}^{M}\right)}{\left(1-P_{b}\left(\zeta \right)\right)}^{L_{Data}}. \end{array}$$

The packets are discarded mainly either due to channel access failure or due to retry limits. Channel access failure occurs, if the tagged device does not find the channel idle during maxMacBackoff number of backoffs. Thus, the probability of channel access failure is

(23) $$\begin{array}{c} P_{cf}=\left\{p+\left(1-p\right)\alpha +\left(1-p\right)\left(1-\alpha \right)\beta \right\}b_{m,0}. \end{array}$$

Considering the maxFrameRetries=r, the probability of transmission failure in *r* attempts is

(24) $$\begin{array}{c} P_{tf}={\left(1-P_{S}\right)}^{r+1} \end{array}$$

Probability of successful packet reception within the maximum number of channel access and retransmission is called as reliability. Hence the reliability is

(25) $$\begin{array}{c} P_{R}=1-P_{cf}-P_{tf} \end{array}$$

### 5.2. Throughput

Normalized throughput is the ratio of average time occupied by the successful transmission to the interval between two consecutive transmissions. Let *S* be the normalized throughput of a device. The channel is sensed busy if at least one device is transmitting data. Let $P_{tr}$ be the probability that there is at least one transmission. Since there are *M* devices attached to the coordinator and *ϕ* is the probability that a device attempts its first CCA, then

(26) $$\begin{array}{c} P_{tr}=\left(1-{\left(1-\phi \right)}^{M}\right)\left(1-\alpha \right)\left(1-\beta \right) \end{array}$$

Hence the normalized throughput of a device is

(27) $$\begin{array}{c} S=\frac{P_{tr}P_{S}L_{D}}{\left(1-P_{tr}\right)\sigma +P_{tr}P_{S}T_{s}+P_{tr}\left(1-P_{S}\right)T_{c}} \end{array}$$

where, $T_{s}$ is the mean time of successful transmission, and $T_{c}$ is the mean time that the channel is occupied because of the collision. $L_{D}$ is the time of payload and *σ* is the duration of a unit backoff slot. $T_{s}$ and $T_{c}$ are calculated as

(28) $$\begin{array}{c} \left\{\begin{array}{cc} T_{s} & =2T_{CCA}+2\sigma +T_{D}+\delta +T_{ACK}\\ T_{c} & =2T_{CCA}+2\sigma +T_{D}+\delta_{max} \end{array}\right\} \end{array}$$

where, $T_{CCA}$, $T_{D}$, $T_{ACK}$, *δ*, $\delta_{max}$ and *σ* are the time duration for performing CCA, transmitting data, receiving an acknowledgement, waiting time for an acknowledgement, maximum waiting time for an acknowledgement and duration of one backoff slot, respectively.

### 5.3. Network Delay

The total time required for the information to be generated by the source device and to be received by the destination device is defined here as the network delay. Let, $D_{j}$ be the delay that the packet is transmitted successfully at time j+1 and the packet has failed for *j* times. Then

(29) $$\begin{array}{cc} D_{j} & =T_{s}+jT_{c}+\sum_{i=0}^{j}D_{b} \end{array}$$

where, $D_{b}$ is the delay that the device successfully found the channel idle during maxMacBackoff number of backoff slots. $T_{s}$ is the mean time of successful transmission and $T_{c}$ is the mean time that the channel is occupied because of the collision. Let $B_{k}$ be the event that the channel access is successful for (k+1)th times and the channel access is failed for *k*th times. Let *B* be the event that the channel access is successful within maxMacBackoff number of backoff. Hence, the expected backoff delay in one transmission attempt is

(30) $$\begin{array}{cc} D_{b} & =\sum_{i=0}^{m}\frac{P_{backoff}^{i}\left(1-P_{backoff}\right)}{1-P_{backoff}^{m+1}}\sum_{k=0}^{i}\frac{W_{i}-1}{2} \end{array}$$

If the packet is generated outside its allocated CAP, then it has to wait until the next allocated CAP comes. Therefore, if the packet arrival follows the Poisson process with packet arrival rate *λ*, then

(31) $$\begin{array}{cc} waiting\;time & =\int_{0}^{BI}\left(BI-x\right)\lambda e^{-\lambda x}dx. \end{array}$$

Propagation delay is the amount of time it takes for the head of the signal to travel from the sender to the receiver. It can be computed as the ratio between the link length and the propagation speed over the specific medium. Let $E_{j}$ be the event that the packet is transmitted successfully j+1 times and the packet has failed for *j* times. Let *E* be the event that the packet is transmitted successfully within maxFrameRetries. Considering the maxFrameRetries=r,

(32) $$\begin{array}{cc} Pr(E_{j}/E) & =\frac{{\left(1-P_{S}\right)}^{j}P_{S}}{1-{\left(1-P_{S}\right)}^{r+1}}. \end{array}$$

Therefore the network delay is

(33) $$\begin{array}{cc} D & =\sum_{j=0}^{r}Pr\left(E_{j}/E\right)D_{j}+waiting\;time+propagation\;delay. \end{array}$$

The packet may not be transmitted in the current CAP period if $D>CAP_{period}$ and has to wait for the next superframe. Therefore, the total average delay is

(34) $$\begin{array}{cc} Delay & =\left\lfloor \frac{D}{CAP_{period}}\right\rfloor BI+D\;mod\;CAP_{period}. \end{array}$$

### 5.4. Energy

In this section, the energy consumption, which is defined as the average energy consumption by a device to transmit one slot amount of data successfully is analyzed. We consider $P_{RX}$ and $P_{TX}$ are the energy consumption to receive and to transmit a packet, respectively. Sensor devices are assumed to be in sleep state during the backoff procedure by which they do not consume any energy. The energy consumption due to turnaround process from the sleeping state to the sensing one is assumed to be $\frac{P_{RX}+P_{TX}}{2}$. Taking $T_{L}$, $T_{A}$, $T_{CCA}$, $T_{ta}$ and $\delta_{max}$ as the time duration of transmitting a packet, receiving an acknowledgement, each successful CCA, turnaround time, maximum time to wait for the acknowledgement, respectively, the total energy consumption per device can be analyzed as follows.

(35) $$\begin{array}{cc} E & =\frac{\alpha T_{CCA}P_{RX}+2\left(1-\alpha \right)\beta T_{CCA}P_{RX}+\left(1-\alpha \right)\left(1-\beta \right)\left\{\left(1-P_{S}\right)E_{c}+P_{S}E_{s}\right\}}{\left(1-\alpha \right)\left(1-\beta \right)P_{S}T_{data}} \end{array}$$

where, $E_{s}$ is the energy consumption of successful transmission, and $E_{c}$ is energy consumption due to collision. $E_{s}$ and $E_{c}$ can be calculated as

(36) $$\begin{array}{c} \left.\begin{array}{cc} E_{s} & =2T_{CCA}P_{RX}+2T_{ta}\frac{P_{RX}+P_{TX}}{2}+T_{L}P_{TX}+T_{A}P_{RX}\\ E_{c} & =2T_{CCA}P_{RX}+2T_{ta}\frac{P_{RX}+P_{TX}}{2}+T_{L}P_{TX}+\delta_{max}P_{RX} \end{array}\right\} \end{array}$$

## 6. Simulation

In this section, we validate and evaluate our models by using OMNeT++ [35] simulator. We perform simulations to compare our proposed schemes with IEEE 802.15.4e. In our simulation, star topology is considered, where each device is deployed randomly around the coordinator. Our work is compared with the IEEE 802.15.4e standard for successful transmission, throughput, reliability, average network delay and energy consumption taking poisson arrival rate. The simulation parameters given in Table 2 are set as per the IEEE 802.15.4e standard. The simulation is performed with 50 runs and the results are given taking average of those 50 runs.

**Figure 6:**
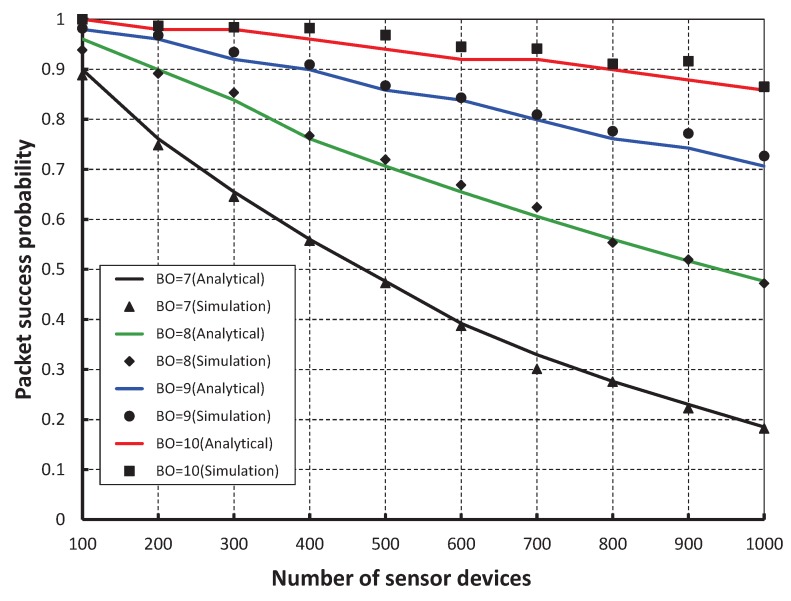
Validation of packet success probability for different beacon order.

As shown in Figure 6, different number of devices are considered to evaluate the corresponding packet success probability. It is observed that the packet success probability decreases as the number of devices attached to a coordinator increases irrespective of the beacon order. When the data payload of 100 bytes and 500 number of devices are considered, the success probability is 0.96, if the beacon order is 10. But, the probability is 0.85, 0.70 and 0.47 for the beacon order 9, 8 and 7, respectively. The difference between the simulation and analytical results varies little due to the distribution of devices to different superframes. The number of associated devices to one CAP decreases by increasing the beacon order.

**Figure 7:**
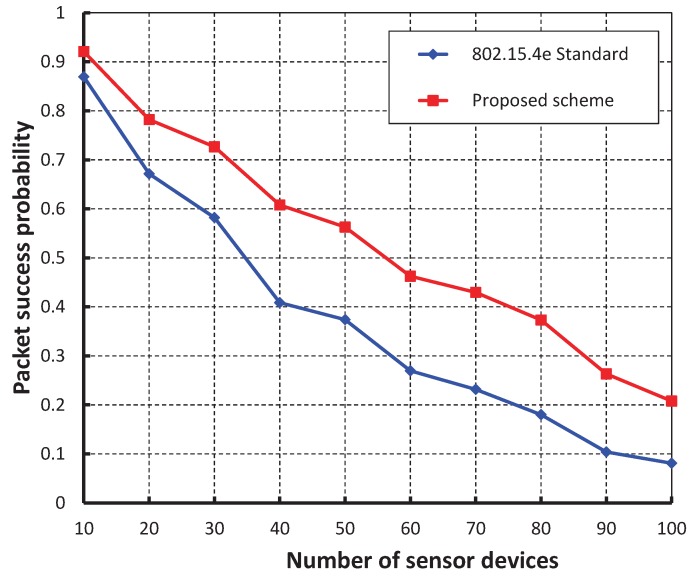
Comparison of packet success probability of our proposed scheme with the IEEE 802.15.4e standard.

**Figure 8:**
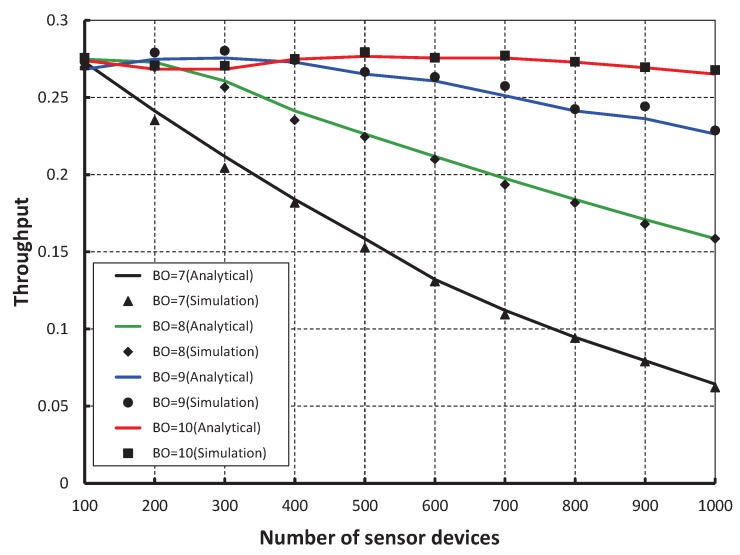
Validation of throughput for different beacon order.

As shown in Figure 7, our work is compared with the IEEE 802.15.4e standard based on the simulation results for different values of beacon order and superframe order. It is observed that the packet success probability of our work is higher as compared to the IEEE 802.15.4e. Figure 8, demonstrates the throughput corresponding to the number of devices. It is observed that the throughput decreases as the number of devices attached to a coordinator increases irrespective of the beacon order. When the data payload of 100 bytes and five hundred number of devices are considered, the throughput is 0.27, if the beacon order is 10.

**Figure 9:**
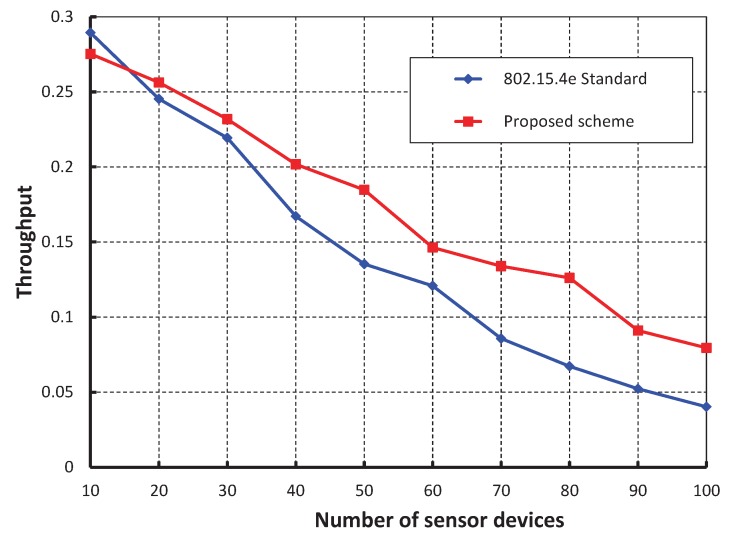
Comparison of throughput of our proposed scheme with the IEEE 802.15.4e standard.

**Figure 10:**
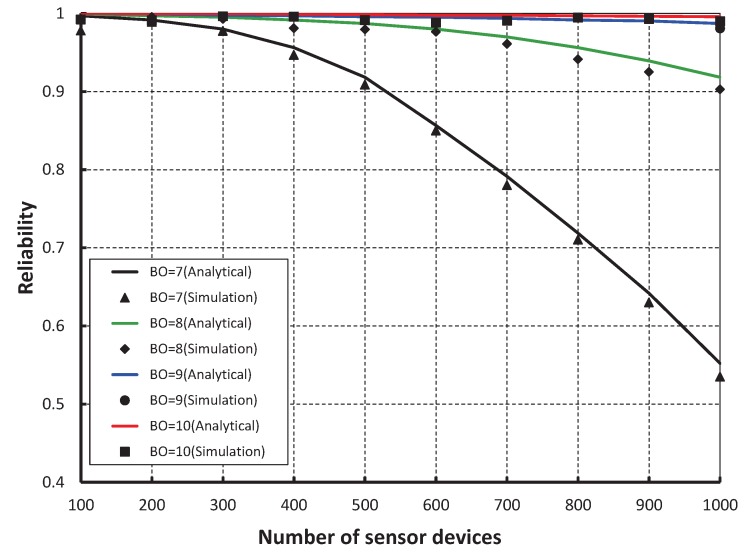
Validation of reliability for different beacon order.

We compare our work with the IEEE 802.15.4e standard for equal number of beacon and superframe order as shown in Figure 9. It is found that the throughput of our scheme is higher than the standard except when the number of devices are less than 15. This happens as we keep two idle slots between CCA1 and CCA2 in our proposed scheme to avoid the channel busy due to acknowledgement. Also, we find that the analytical result well match with the simulation one. The reliability of data transmission with respect to different numbers of devices is compared as shown in Figure 10. It is observed that the reliability decreases as the number of devices attached to a coordinator increases irrespective of the beacon order. When the data payload of 100 bytes and 500 number of devices are considered, the reliability becomes 0.99 when beacon order is 10. However, it decreases when the value of beacon order becomes 9, 8 and 7.

**Figure 11:**
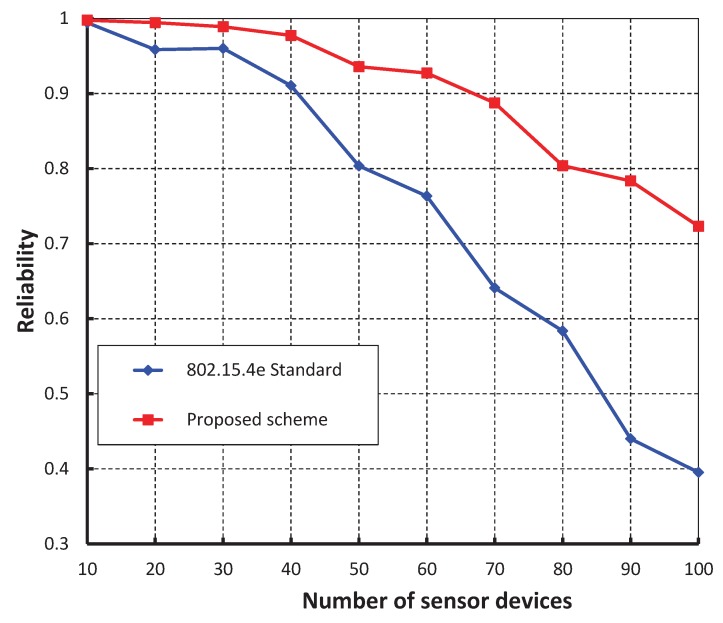
Comparison of reliability of our proposed scheme with the IEEE 802.15.4e standard.

**Figure 12:**
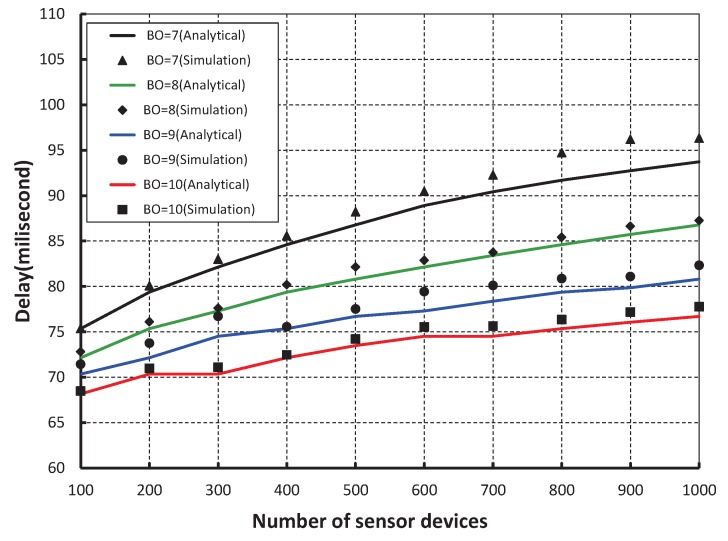
Validation of packet delay for different beacon order.

As shown in Figure 11, our work is fairly compared with the IEEE 802.15.4e standard maintaining equal beacon and superframe order. It is observed that the reliability of our scheme is higher and the simulation result matches with the analytic one. Figure 12, demonstrates the delay corresponding to the number of devices. It is realized that the delay increases as the number of devices increases irrespective of the beacon order. When the data payload of 100 bytes and 500 sensor devices are considered, the delay is nearly 74 ms, if the beacon order is 10.

**Figure 13:**
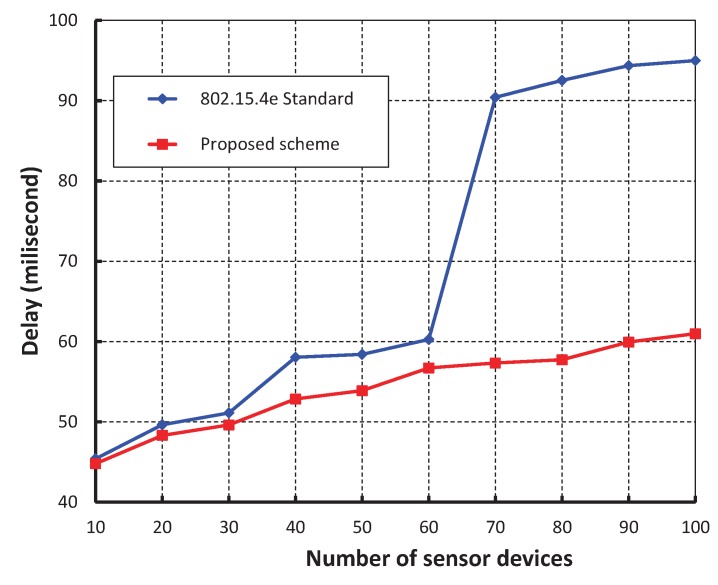
Comparison of packet delay of our scheme with IEEE 802.15.4e standard.

**Figure 14:**
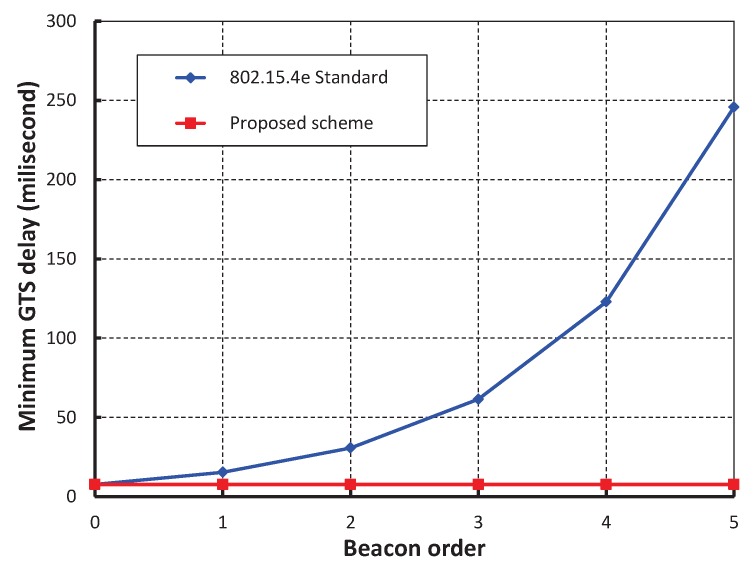
Comparison of GTS retransmission delay of our scheme with IEEE 802.15.4e standard.

**Figure 15:**
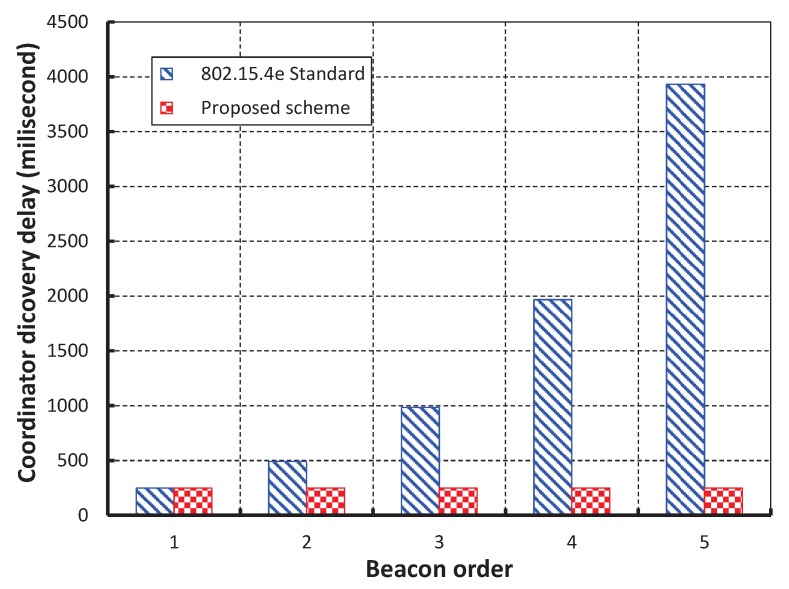
Comparison of minimum coordinator discovery delay of our proposed scheme with the IEEE 802.15.4e standard.

We have compared our work with IEEE 802.15.4e standard setting beacon and superframe orders as 2 as shown in Figure 13. It is noticed that delay of our work is significantly less and the simulation result matches with the analytic one. This happens as our proposed method avoids the unnecessary backoff durations. The delay suddenly increases in case of IEEE 802.15.4e when the number of devices reaches to 70. This situation occurs due to channel access failure or transmission failure by a device and the device has to wait for the start of the next superframe. It is to be noted that the device has to wait for the CFP period before accessing the channel again. However, in our protocol, the chance of accessing the channel in the next superframe is significantly less due to the high transmission success rate as compared to the IEEE 802.15.4e. Figure 14, shows the minimum GTS packet delay with respect to different numbers of beacon order. It is observed that the minimum GTS retransmission delay remain constant irrespective of the beacon order. This happens as the MAC scheme of our scheme allows the failed devices to retransmit data during the CAP without waiting for the next allotted retransmission slots. As shown in Figure 15, we compare our scheme with IEEE 802.15.4e standard taking beacon order from 1 through 5 and superframe order as 1. It is found that coordinator discovery delay of our scheme remains constant though it exponentially increases in case of IEEE 802.15.4e. This happens as the coordinator broadcasts the beacon sequentially in every channel in our proposed scheme.

**Figure 16:**
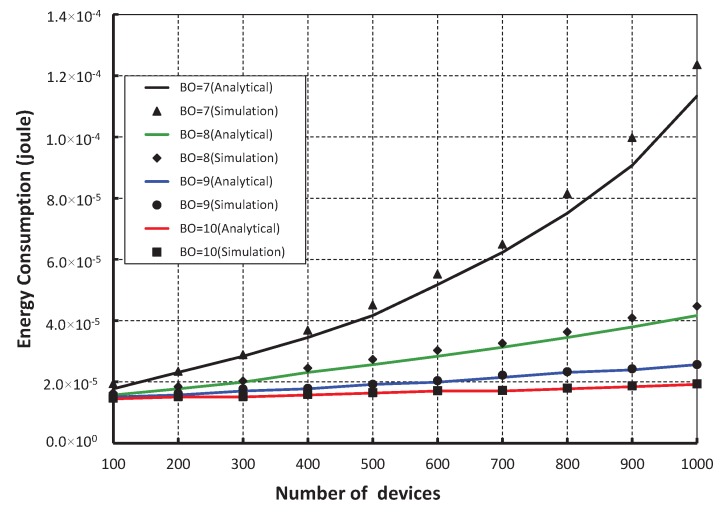
Validation of energy consumption for different beacon order.

**Figure 17:**
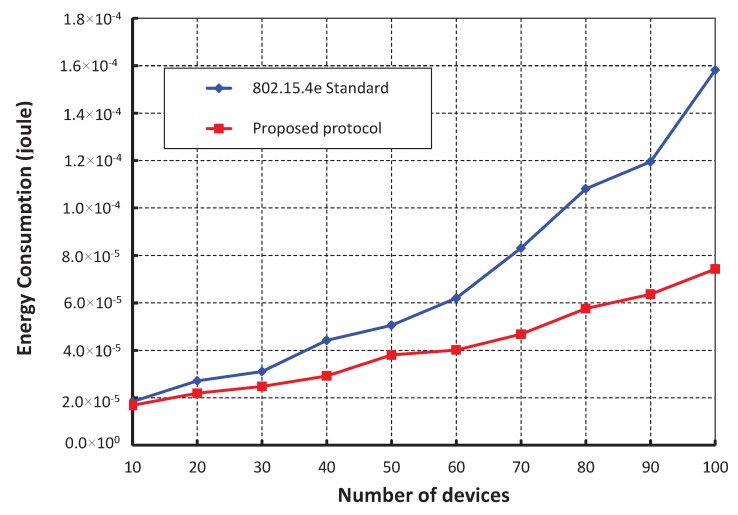
Comparison of energy consumption of our scheme with IEEE 802.15.4e standard.

As shown in Figure 16, different numbers of devices are considered to evaluate the corresponding energy consumption in joule. It is observed that the energy consumption increases as the numbers of devices attached to a coordinator increase irrespective of the beacon order. When the data payload of 100 bytes and five hundred number of devices are considered, the energy consumption is nearly 1.64 × 10^−5^ joule, if the beacon order is 10. As shown in Figure 17, our work is compared with IEEE 802.15.4e standard for equal number of beacon and superframe order. It is found that the energy consumption of our scheme is less as compared to the IEEE 802.15.4e standard. This happens as the numbers of sensors in our scheme are restricted to access the channel within one CAP by which number of collisions is reduced significantly. Besides, the simulation result of our scheme matches with the analytic one.

## 7. Conclusions

In this paper, a new channel access scheme and a beacon broadcast scheme are proposed to avoid the collision in dense wireless network with mobile devices. A new dynamic guaranteed retransmission slot allocation scheme is proposed to reduce the GTS retransmission delay, which is reduced significantly as compared to the IEEE 802.15.4e standard. Analytical models are designed and performance analyses are made for the data transmission reliability, throughput, energy consumption and success rate probability based on our proposed schemes. Our models are validated with the simulation and analytical methods, which are well matched and comparisons of our schemes are made with IEEE 802.15.4e standard. The results obtained from the analytical and simulation show that our schemes can improve the reliability, throughput, delay and energy consumptions significantly.

## Figures and Tables

**Table 1:** Notation table.

Notation	Meaning
*M*	Total number of devices assigned to one superframe;
$L_{D}$	Length of a data packet;
$L_{ACK}$	Length of an acknowledgement packet;
*q*	Packet generation probability;
$R_{b}$	Duration of random backoff;
*p*	Probability that remaining time slots are sufficient to transmit a packet;
*ϕ*	Probability that a device is sensing the channel first time;
*α*	Probability that the channel is busy during first CCA;
*β*	Probability that the channel is busy during second CCA;
$P_{b}\left(\zeta \right)$	Probability of channel bit error rate;
$P_{S}$	Probability of transmission success;
$P_{C}$	Probability of transmission collision;
$P_{tr}$	Probability of transmission;
$T_{CCA}$	Duration of CCA;
$T_{D}$	Duration of data transmission;

**Table 2:** Simulation parameters.

Parameters	Value
Radio band	2.4 GHz
Channel bandwidth	250 kbps
Carrier sense sensitivity	−85 dBm
Channel number	11∼26
Beacon interval	15.6× 2^4^ ms
Unit backoff period	20 symbol
PHY overhead	6 byte
MAC overhead	3 byte
Transmission current consumption	9.1 mA
Receiving current consumption	5.9 mA
Turnaround current consumption	7.5 mA
Sleep current consumption	0.001 mA
